# High prevalence of obesity among women who enrolled in HIV prevention trials in KwaZulu-Natal, South Africa: healthy diet and life style messages should be integrated into HIV prevention programs

**DOI:** 10.1186/1471-2458-13-159

**Published:** 2013-02-21

**Authors:** Handan Wand, Gita Ramjee

**Affiliations:** 1 The Kirby Institute, University of New South Wales, Sydney, NSW 2052, Australia; 2 HIV Prevention Research Unit, Medical Research Council, Durban, South Africa

**Keywords:** Body mass index, Sexual risk behaviours, HIV, South Africa

## Abstract

**Background:**

In South Africa, poverty and the dual epidemics of HIV and tuberculosis underscore the need for prevention efforts for obesity. The aim of this study was to describe the prevalence of obesity in a cohort of South African women and discuss the implications for public health practices.

**Methods:**

A total of 5,495 HIV-negative women from KwaZulu-Natal, South Africa enrolled in three microbicide trials during the period of 2002–2008 were categorised as normal weight (body mass index (BMI: 18.6-<25), overweight (BMI: 25-<30) or obese (BMI: 30+). Incidence of HIV and other sexually transmitted infections such as *Chlamydia* and gonorrhoea were also estimated and compared by BMI groups. Combined data was analysed using STATA 10.0.

**Results:**

Approximately 70% of the sample population was classified as being overweight or obese. Older age and lack of education were determined to be *significant predictors of* obesity. Women who were 35 years or older were more than three times as likely to be overweight and more than 12 times as likely to be obese compared to the youngest group. The highest HIV and STI incidence rates were observed among those with BMI <25 kg/m^2^ (normal weight) compared to women with BMI more than 25 kg/m^2^ (8.1 and 19.8 per 100 person-year respectively, P<0.001, both).

**Conclusion:**

Effective obesity prevention strategies are needed to re-formulate HIV prevention programmes by incorporating healthy diet and life style messages to target those who are at highest risk not just for HIV infection but also for non-communicable diseases.

## Background

Obesity plays a major role in chronic non-communicable diseases, including type-2 diabetes, cardiovascular disease (CVD), and hypertension [1]. Globally, 1.3 billion people are estimated to be overweight or obese [2]. Obesity is not only affecting developed countries but is becoming an increasing problem in developing countries [3-5]. There has been a progressive increase in the prevalence of obesity in South Africa, particularly among women and young girls [6-8]. A report published by the South African Medical Research Council (MRC) in 2007, cited that “56% of adult women and 29% of adult men were overweight/obese, while 17% of children under the age of 9 years were overweight” [7,9]. The MRC also reported that “approximately 60 people die from obesity-related disease every day in South Africa”. The primary reason for these high figures, particularly among black women, may be a number of misconceptions regarding the health risk of obesity, including the strong belief that increased body weight is an indication of a good state of health [10,11]. In addition, research conducted in the US indicates that access to and availability of nutritionally balanced food is associated with socio-economic status and structural factors, including the absence of accessible retailers of quality food in economically depressed or rural areas [12]. Similar phenomena could also be relevant to the South African situation.

South Africa is a developing country with a high prevalence of poverty [13]. Other factors such as violence, lack of education and inadequate services contribute to the increasing number of cases of non-communicable as well as communicable diseases [14,15]. South Africa bears the largest global burden of the HIV epidemic, with an estimated population of 5.7 million individuals living with the virus [16], and the third highest number of incident tuberculosis cases. The prevalence of obesity, particularly among black South African women, reflects globalisation, which is the primary driving force behind the changes in healthy diet and life style (i.e. nutritional transition) [17]. Developing and implementing obesity prevention programs will present numerous challenges in many South African communities where HIV, tuberculosis and poverty are pervasive, and access to a nutritionally balanced food supply is limited [3].

The HIV Prevention Research Unit of the Medical Research Council in Durban, KwaZulu-Natal, has been involved in several biomedical intervention clinical trials in HIV prevention. It has recruited more than 15,000 women. Counselling sessions associated with these trials educate women on how to protect themselves against HIV infection [18,19]. However, healthy diet and life style messages have never been incorporated into these programs.

The primary aim of this paper was to estimate the prevalence of obesity among a cohort of women, who enrolled in one of the three biomedical intervention trials during the period of 2002–2008 at clinical research sites in KwaZulu-Natal, South Africa. We also compared HIV seroconversion rates and other sexually transmitted infections across the women who were classified as normal, overweight and obese. Therefore implementing healthy diet and life style messages into these programs will create a unique opportunity (without an extra cost) to target those who are at most risk for obesity.

## Methods

### Participants and design

The current study included data derived from three biomedical intervention trials. Details of the main trials have been published elsewhere [20-22]. The present analysis included 1,485 women who enrolled in the Methods for Improving Reproductive Health in Africa (MIRA) trial of the diaphragm for HIV prevention (2002–2005). The Microbicides Development Programme (MDP) 301 study was a multi-centre, phase III, randomized, double-blind, and parallel group trial of a vaginal microbicide (2005–2008); we included 2,554 women who enrolled in MDP 301. The candidate microbicide Carraguard (PC-515), developed by the Population Council, was a non-contraceptive vaginally applied gel. Its efficacy against HIV infection was investigated in a randomized, placebo-controlled, double-blind, phase III trial (2004–2007). The present analysis included data from 1,456 women who enrolled in the Carraguard trial.

All protocols and informed consent forms were approved by the Biomedical Research Ethics Committee at the University of KwaZulu-Natal, as well as the various study-specific Institutional Review Boards.

### Measurements

The majority of the demographic information was collected by face-to-face interview. Body mass index (BMI) (kg/m^2^) was calculated based on clinically assessed weight (kg) and height (m) at baseline and month 12. The current analysis considered only the baseline measurements as there was no notable change with regard to BMI during the follow up across the trials. WHO definitions were used to categorize women as normal weight (BMI: 18.5–24.9), overweight (BMI: 25–29.9) and obese (BMI: 30 and above). Age was stratified into 4-year categories: 18–24 years, 25–29 years, 30–34 years, and 35+ years. Women were also categorized based on their self-reported level of education (less than high school vs. at least high school or higher) and employment status (regular income vs. none/irregular income).

### Statistical analyses

Demographic and sexual history variables were described by BMI groups. We used logistic regression models to test for the effect of age groups, level of education and employment status in obese (versus normal weight) and overweight (versus normal weight) women separately.

Kaplan-Meier survival analyses were conducted to estimate the proportion of women acquiring HIV infection and STIs during the study. Seroconversion date was estimated as the midpoint between the first visit date at which the woman tested positive for HIV and the previous negative test date. Time to seroconversion was defined as the difference between the seroconversion date and the enrolment date.

Cox proportional hazard regression models were used to determine the association between baseline BMI and incidence of HIV and STIs. We also reported age adjusted analysis; however, since the primary sexual risk factors were not collected in a consistent way it was not possible to combine them across the trials. Therefore we did not perform multivariate analyses in this combined population.

All analyses were conducted using STATA 10.0 (College Station, TX, USA).

## Results

Respondents were described overall and across the BMI categories in terms of age, level of education and employment status (Table 1). Of 5,495 women, 1,790 (32%) had a BMI less than 25 (normal weight), 1,739 (32%) had a BMI between 25 and 29 (overweight) and 1,966 (36%) had a BMI of 30 or more (obese). Overall, the median age was 27 (inter-quartile range [IQR]:22–37). The median age in the three BMI weight groups (normal weight, overweight, and obese) were 22 (IQR: 20–28), 27 (IQR: 22–36), and 34 (IQR: 26–40) years, respectively. Proportions of women reported completing high school were 36%, 30% and 23% for normal weight, overweight and obese women respectively; employment rates were reported as 15%, 20% and 22% for normal weight, overweight and obese respectively.

**Table 1:** Distribution of demographics by (body mass index) BMI groups

	**Total N=5,495**	**Normal weight (BMI:18.6-24.9) N=1,790**	**Overweight (BMI:25.0-29.9) N=1,739**	**Obese (BMI: 30+) N=1,966**	**Odds ratio (95% CI)**	
					**Overweight versus normal**	**Obese versus normal**
All women	100%	32%	32%	36%	-	-
**Age, median (IQR)**	27 (22–37)	22 (20–28)	27 (22–36)	34 (26–40)	-	-
<25	2203 (40%)	1,147 (64%)	694 (40%)	362 (18%)	1	1
25-29	908 (16%)	273 (15%)	316 (18%)	319 (16%)	1.91 (1.59,2.31)	3.70 (3.03,4.52)
30-34	700 (13%)	129 (7%)	241 (14%)	330 (17%)	3.09 (2.44,3.90)	8.11 (6.41,10.26)
35+	1684 (31%)	241 (13%)	488 (28%)	955 (49%)	3.35 (2.79,4.01)	12.56 (10.44,15.10)
**Education level**						
At least high school	1649 (30%)	644 (36%)	522 (30%)	452 (23%)	1	1
Less than high school	3846 (70%)	1146 (64%)	1217 (70%)	1514 (77%)	1.47 (1.27,1.70)	1.59 (1.38,1.83)
**Employment status**						
Yes	1044 (19%)	161 (15%)	348 (20%)	433 (22%)	1	1
No	4451 (81%)	1529 (85%)	1391 (80%)	1533 (78%)	1.40 (1.14,1.72)	1.61 (1.32,1.96)

The data showed a significant age effect (Table 1). Women who were 35 years or older were more than three times as likely to be overweight and more than 12 times as likely to be obese compared to the youngest group (i.e. <25 years) [Odds Ratio (OR): 3.35, 95% Confidence Interval (CI): 2.79-4.01 and OR:12.56, 95% CI: 10.44-15.10 respectively]. Women who had less than high school education were approximately 50% more likely to be overweight or obese compared to those who had completed high school (OR: 1.47, 95% CI:1.27-1.70 and OR: 1.59, 95% CI: 1.38-1.83). Overweight and obese women were more likely to report a regular income compared to women of normal weight (OR: 1.40, 95% CI: 1.14-1.72 and OR: 1.61, 95% CI: 1.32-1.96 respectively).

HIV and sexually transmitted infection incidence rates were determined by BMI group during the study (Table 2). The highest HIV and STI incidence rates were observed among those with BMI <25 kg/m^2^ (normal weight) (8.1 and 19.8 per 100 person-year respectively) compared to women with BMI more than 25 kg/m^2^ (P<0.001, both). Adjusted and unadjusted hazard ratios (HR) and associated 95% Confidence Intervals (CIs) are presented in Table 2. After adjusting for age, only those with a baseline BMI 25–29 kg/m^2^ were more likely to HIV seroconvert compared to those in the BMI 30 kg/m^2^ or more. The estimated relative risk of STIs during the trial was also progressively higher in the overweight and normal weight group compared to obese women.

**Table 2:** Incidence of HIV infection and sexually transmitted infections by BMI

**At enrolment**	**HIV incidence (6.7 per 100 person year, 95% CI: 6.1-7.4 per 100 person year)**					
	**Crude incidence rate per 100 Person Year (95% CI)**	**p-value**^**†**^	**Unadjusted Hazard Ratio (95% CI)**	**p-value**	**Adjusted**^**‡**^**Hazard R (95% CI)**	**p-value**
**Body Mass Index (kg/m**^**2**^**)**		<0.001				
Obese^1^	4.6 (3.8,5.6)		1		1	
Overweight^2^	7.8 (6.7,9.0)		1.70 (1.33,2.17)	<0.001	1.36 (1.06,1.75)	0.015
Normal^3^	8.1 (7.0,9.4)		1.76 (1.38,2.25)	<0.001	1.19 (0.92,1.55)	0.185
		**STI**^‡‡^**incidence (15.6 per 100 person year, 95% CI:14.6-16.6 per 100 person year)**				
**Body Mass Index (kg/m**^**2**^**)**		<0.001				
Obese^1^	10.9 (9.6,12.3)		1		1	
Overweight^2^	16.6 (15.0,18.4)		1.82 (1.55,2.14)	<0.001	1.46 (1.22,1.75)	<0.001
Normal^3^	19.8 (17.9,21.9)		1.55 (1.31,1.83)	<0.001	1.40 (1.18,1.66)	<0.001

^†^ P value for log rank test.

^‡^ Age adjusted.

^‡‡^*Chlamydia trachomatis* and gonorrhoea.

^1^ 30+ BMI, ^2^ (25-<30) BMI, ^3^ (18.6-<25) BMI.

## Discussion

Our study confirms an extremely high prevalence of overweight or obesity in this reasonably large cohort of women. Approximately 70% of the women were categorized as being overweight or obese indicating an alarming health concern in this population. This is slightly higher than overall figures reported for South Africa, and is the highest found among other African countries [6-9].

Our study found approximately 50% of the obese women were older than 35 years of age. This strong link between obesity and age suggests that it might be vital to discuss healthy diet and body weight issues at the same time as issues of sexuality and sexual risk with young women, before they start gaining weight at older ages.

It is important to address the steady rise in the prevalence of obesity in South African women. In South Africa, poverty and the twin epidemics of HIV and tuberculosis mean that prevention efforts for obesity may not have the highest priority. However, evidence suggests that, just like in the developed world, obesity and its co-morbidities are adversely affecting the lives of South Africans and the consequent burden of disease contributes to the increasing cost of health care. We recommend identifying effective obesity prevention strategies and integrating them into HIV prevention and management programmes as a component of the interventions needed to enhance the overall health of the population. Implementing healthy diet and life style messages into upcoming HIV/AIDS prevention programs will be beneficial without an extra cost. However, large scale prevention efforts are needed to help control the epidemic. Recently, a survey (The South African National Health and Nutrition Examination Survey – SANHANES-1) was planned for 2011–2012 to evaluate the health status of South Africans for non-communicable diseases, particularly those associated with diet and life style [23]. Results from the SANHANES-1 will inform the size of the problem among South African men and women.

Our study has several limitations. First, the data used in this study were taken from a cohort of women who participated to HIV prevention trials. Therefore, there may be selection bias of women willing to engage in clinical trials such as the ones included in this study. Second, primary sexual risk factors were measured differently at different time points across the trials considered in this study. Therefore, we did not assess the variables that may have explained the relationship between body weight and sexual risk. Consequently, it is possible that being overweight and being at risk for HIV or STIs are due to unmeasured covariates which is not the scope of the current study. Finally, information on nutritional and/or physical activity status was not collected in these trials.

 Nevertheless, this study found significant associations; the hazard ratios demonstrated reasonably large effect sizes indicating a strong signal regarding these associations. Finally, since this is a secondary analysis, we did not determine statistical power a prior to the study. Well-designed studies with adequate power are necessary to answer the predictors of the obesity among women in South Africa.

## Conclusions

Current study presented evidence for unacceptable high rates of obesity among South African women and our study adds new evidence using data from more than 5,000 women; while while this group is at higher risk for non-communicable diseases compared to women of normal weight, normal weight women were at increased risk for communicable diseases compared to those who were overweight or obese. New strategies are needed to re-formulate existing HIV prevention and treatment programs by incorporating information on a healthy diet and lifestyle to produce holistic health and wellness interventions.

## Competing interests

The authors declare that they have no competing interests.

## Authors’ contributions

Contributors GR was the principal investigator of all the trials from Durban clinical research sites. HW performed the statistical analysis. GR and HW interpreted and drafted the manuscript. Both authors read and approved the final manuscript.

## Pre-publication history

The pre-publication history for this paper can be accessed here:

http://www.biomedcentral.com/1471-2458/13/159/prepub
